# Ectodomain Shedding by ADAM17: Its Role in Neutrophil Recruitment and the Impairment of This Process during Sepsis

**DOI:** 10.3389/fcimb.2017.00138

**Published:** 2017-04-25

**Authors:** Hemant K. Mishra, Jing Ma, Bruce Walcheck

**Affiliations:** Department of Veterinary and Biomedical Sciences, University of MinnesotaSt. Paul, MN, USA

**Keywords:** adhesion, leukocyte, inflammation, infection, bacteria

## Abstract

Neutrophils are specialized at killing bacteria and are recruited from the blood in a rapid and robust manner during infection. A cascade of adhesion events direct their attachment to the vascular endothelium and migration into the underlying tissue. A disintegrin and metalloproteinase 17 (ADAM17) functions in the cell membrane of neutrophils and endothelial cells by cleaving its substrates, typically in a *cis* manner, at an extracellular site proximal to the cell membrane. This process is referred to as ectodomain shedding and it results in the downregulation of various adhesion molecules and receptors, and the release of immune regulating factors. ADAM17 sheddase activity is induced upon cell activation and rapidly modulates intravascular adhesion events in response to diverse environmental stimuli. During sepsis, an excessive systemic inflammatory response against infection, neutrophil migration becomes severely impaired. This involves ADAM17 as indicated by increased levels of its cleaved substrates in the blood of septic patients, and that ADAM17 inactivation improves neutrophil recruitment and bacterial clearance in animal models of sepsis. Excessive ADAM17 sheddase activity during sepsis thus appears to undermine in a direct and indirect manner the necessary balance between intravascular adhesion and de-adhesion events that regulate neutrophil migration into sites of infection. This review provides an overview of ADAM17 function and regulation and its potential contribution to neutrophil dysfunction during sepsis.

## Neutrophils

**Figure 1:**
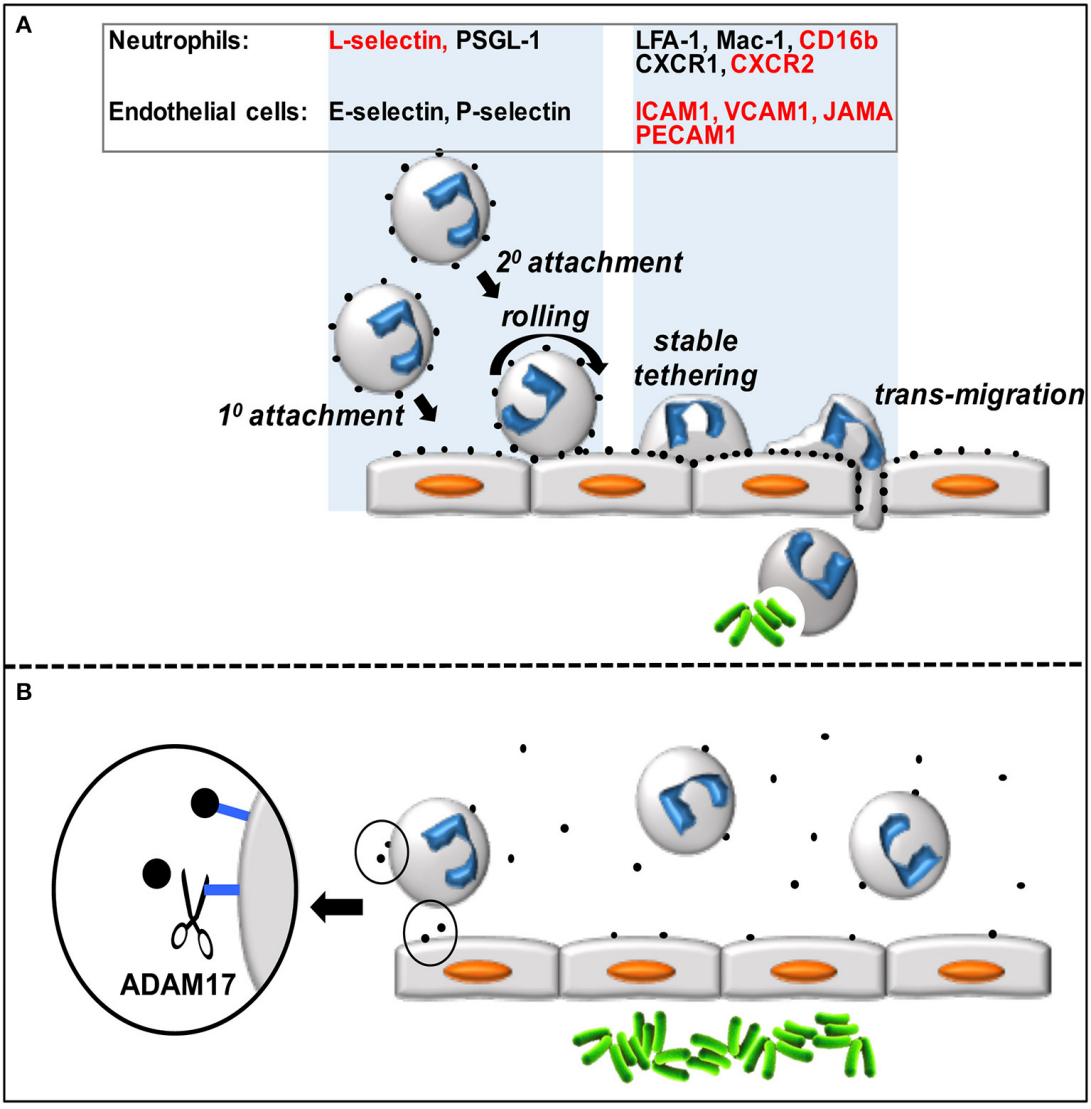
**(A)** Circulating neutrophils attach to and transmigrate through the vascular endothelium in a step-wise process. Neutrophils accumulate on the vascular endothelium by direct (1°) and indirect (2°) manners, roll and scan the endothelial cells for chemokines, which promotes stable tethers and eventual transmigration into the underlying tissue. Various neutrophil and endothelial cell adhesion molecules and receptors directly involved in this process (represented by black dots) are listed in the figure, and those that are ADAM17 substrates are indicated in red. **(B)** Over-activation of ADAM17 by inflammatory stimuli during sepsis may result in excessive ectodomain shedding by neutrophils and endothelial cells that in turn impairs neutrophil recruitment and bacterial (green rods) clearance.

Neutrophils are the predominant leukocyte population in the blood of healthy individuals and serve a critical function in host protection and wound healing, as described by others in this research topic and in recent reviews (Kolaczkowska and Kubes, 2013; Mayadas et al., 2014). These innate immune cells are produced in the bone marrow and reside in the blood where they are poised for a rapid influx into sites of acute inflammation. Neutrophils are professional phagocytes that engulf bacteria and kill them through the release of lytic enzymes and reactive oxygen species. They can also impede the spread of extracellular pathogens through the production of neutrophil extracellular traps. Circulating neutrophils infiltrate sites of inflammation by an exquisitely orchestrated multistep adhesion cascade (Figure 1A) (Kolaczkowska and Kubes, 2013). The first step is their attachment to vascular endothelial cells (e.g., lining post-capillary venules) that have been activated by events in the underlying tissue. The loosely attached neutrophils are pushed along by the blood flow, causing them to roll, and survey the luminal surface of endothelial cells for chemokines that will promote their stimulation and more stable attachment and transmigration through the vascular wall. Neutrophil attachment and rolling is primarily mediated by selectin adhesion proteins (L-selectin on neutrophils and E- and P-selectin on activated endothelial cells) that recognize various mucin-like molecules, such as PSGL1. In addition to free-flowing neutrophils attaching directly to endothelial cells (referred to as 1° or direct attachment), they can also attach to other neutrophils that have already accumulated on the vascular endothelium (2° or indirect attachment) (Figure 1A). The latter process is mediated by L-selectin and PSGL1 (Walcheck et al., 1996b), and has been shown *in vitro* and *in vivo* to amplify neutrophil accumulation (Bargatze et al., 1994; Walcheck et al., 1996b; Sperandio et al., 2003; St. Hill et al., 2003). Indeed, neutrophil infiltration into inflamed tissues occurs in a prodigious manner and has been referred to as “swarming” based on *in vivo* imaging (Lämmermann, 2016).

Neutrophils attached to the vascular endothelium transition from rolling to firm adhesion upon their stimulation by chemokines, which induce a high affinity state by integrin adhesion proteins, such as LFA-1 and Mac-1. These integrins bind to the immunoglobulin superfamily members ICAM1 and ICAM2 that are upregulated in expression by activated endothelial cells. Neutrophil transmigration across the vascular wall also involves VCAM1, PECAM1, and JAMA. Upon entering the underlying tissue, neutrophils move in a directed manner, guided by a hierarchy of chemotactic factors, to the origin of pathogen and damaged cell-associated molecular patterns (PAMPs and DAMPs). The primary chemokine receptors expressed by human neutrophils involved in promoting their firm adhesion to the vascular wall and chemotaxis are CXCR1 (binds to CXCL6 and CXCL8) and CXCR2 (binds to CXCL1-3 and CXCL5-8) (Sadik et al., 2011). CXCR2 has been extensively examined in animal models as well (Stadtmann and Zarbock, 2012), and on mouse neutrophils this receptor binds to KC, MIP-2, and LIX (Cacalano et al., 1994; Goncalves and Appelberg, 2002; Sadik et al., 2011).

## Neutrophil dysfunction during sepsis

Sepsis is a severe systemic inflammatory response to microbial pathogens (primarily bacterial and to a lesser degree fungal or viral), and is the primary cause of death from infection (Cohen et al., 2015). Since the early 1990s, this disorder was defined by using four categories; systemic inflammatory response syndrome, sepsis, severe sepsis, and septic shock. Due to increased scientific understanding of sepsis pathophysiology, the definition of the sepsis syndrome has been recently updated to just *sepsis*, defined as “life-threatening organ dysfunction due to a dysregulated host response to infection,” and *septic shock*; “a subset of sepsis where underlying circulatory and cellular/metabolic abnormalities are profound enough to substantially increase mortality” (Singer et al., 2016).

The Surviving Sepsis Campaign (www.survivingsepsis.org) established standards for the diagnosis and management of sepsis, and this has led to decreases in early mortality (Dellinger and Vincent, 2005; Kumar et al., 2011). However, epidemiologic studies reveal that the incidence of sepsis is still on the rise, and this will likely continue as the general population ages, as immune compromising therapies for cancer and autoimmune disease become more prevalent, and as microbial antibiotic resistance increases. Remarkably, current estimates indicate that 1 million people with sepsis are hospitalized per year in the US and >30 million globally (Liu et al., 2014; Cohen et al., 2015). According to the Healthcare Cost and Utilization Project by the U.S. Department of Health & Human Services, Agency for Healthcare Research and Quality, sepsis is the most expensive condition treated in US hospitals (www.hcup-us.ahrq.gov).

Sepsis is initiated by the innate immune system's recognition and response to PAMPs and DAMPs. This response greatly affects immune homeostasis, with an acute phase that is both pro- and anti-inflammatory and a secondary phase in which the adaptive immune system is suppressed. The intensity and duration of these responses are associated with increased secondary infections and mortality (Gotts and Matthay, 2016). It is well–established in animal models subjected to sepsis and by clinical evidence in humans that circulating neutrophils become activated, which impairs their migration to sites of infection and causes them to sequester in the vascular beds of organs where they promote vascular occlusions and leakage, and tissue destruction (Alves-Filho et al., 2010b; Sônego et al., 2014; Lerman and Kim, 2015). These are key events that promote multiple organ failure and septic shock.

The multistep adhesion cascade by which circulating leukocytes infiltrate sites of inflammation requires rapid orchestration of adhesion and de-adhesion events. A critical mechanism that underpins this process is ectodomain shedding, which is the focus of this review. There is increasing evidence for aberrant regulation of ectodomain shedding during inflammatory disorders and its association with vascular dysfunction during sepsis (Gearing and Newman, 1993; Cowley et al., 1994; Muller Kobold et al., 1998; Zonneveld et al., 2014; Lerman and Kim, 2015).

## Ectodomain shedding

Ectodomain shedding is a proteolytic process in which cell surface proteins are cleaved at an extracellular location proximal to the cell membrane, resulting in the release of an intact ectodomain and the retention of a membrane-associated fragment (Weber and Saftig, 2012). Cleaved proteins include many type I and type II transmembrane proteins and some glycosylphosphatidylinositol (GPI)-linked proteins. Cell surface proteins that are shed have diverse functions and include adhesion molecules, cytokines, chemokines, growth factors, and their receptors (Scheller et al., 2011). The shedding process of these substrates regulates the density of cell surface receptors, the release of factors that serve as agonists, and the release of soluble receptors that can function as antagonists.

**Figure 2:**
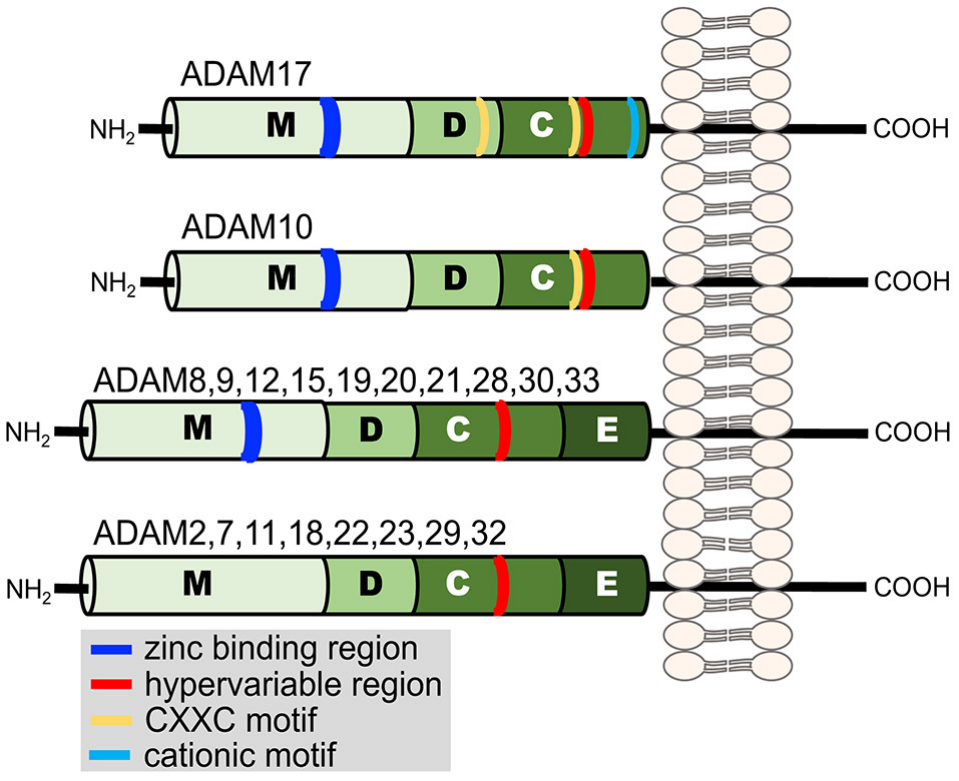
**Illustration of the domain structure of the human ADAM family members**. Each domain is indicated by a letter. Metalloproteinase (M), disintegrin-like (D), cysteine-rich (C), and epidermal growth factor (E). Additional regions of functional relevance discussed in the text are indicated in the key.

Ectodomain shedding primarily occurs by a disintegrin and metalloproteinases (ADAMs) and to a lesser degree by matrix metalloproteinases (MMPs), members of the adamalysin and matrixin subfamilies, respectively, of the metzincin metalloproteinase superfamily (Khokha et al., 2013). Metzincin derives its name from the conserved methionine amino acid adjacent to a zinc-binding motif in the catalytic region of the proteases. The ADAMs are type-1 transmembrane proteins with distinct modular domains consisting of, from N- to C-terminus, a metalloproteinase domain, disintegrin-like domain, cysteine-rich domain, an epidermal growth factor domain (note ADAM10 and 17 lack this domain), a transmembrane segment, and a cytoplasmic region (Figure 2) (Takeda, 2016). Twenty ADAMs have been identified in humans, excluding pseudogenes, and of these only 12 are proteolytically active (ADAM8, 9, 10, 12, 15, 17, 19, 20, 21, 28, 30, and 33) (Weber and Saftig, 2012; Hartmann et al., 2013; Takeda, 2016).

ADAM17 and ADAM10 are the most similar in terms of amino acid sequence and structure (Rosendahl et al., 1997; Maskos et al., 1998), and at this time they are the most widely studied. Though ADAM17 and ADAM10 are not redundant sheddases (Jones et al., 2016), there is some overlap in their substrate repertoire, which may serve a compensatory role and/or enable differential shedding of common substrates. Indeed, ADAM10 has been reported to function primarily in a constitutive manner, whereas ADAM17 is highly inducible, responding to various cellular stimuli (Le Gall et al., 2009), as described in more detail below. ADAM17's role in neutrophil effector functions has been broadly examined (Li et al., 2006; Walcheck et al., 2006; Bell et al., 2007; Chalaris et al., 2007; Horiuchi et al., 2007a; Schaff et al., 2008; Wang et al., 2009, 2010, 2011, 2013; Long et al., 2010, 2012; Arndt et al., 2011; Scheller et al., 2011; Tang et al., 2011; Mishra et al., 2015, 2016), and is discussed below.

## ADAM17

Approximately 20 years ago, Roy Black's group and others provided direct evidence that ADAM17 converts transmembrane TNFα to its soluble form (Black et al., 1997; Moss et al., 1997). Soon afterwards this group also demonstrated through ADAM17 gene inactivation in mice that the sheddase had a much broader role than inflammation regulation and was essential for mammalian development due to EGFR ligand cleavage and EGFR signaling (Peschon et al., 1998). Global deletion of the *Adam17* gene in mice is predominantly perinatal lethal (Peschon et al., 1998; Horiuchi et al., 2007a), though the degree of lethality depends on the background of the mouse strain (Li et al., 2007). However, mice expressing severely reduced levels of ADAM17, due to spontaneous or induced mutations of its gene, demonstrate significantly increased survival when compared to total inactivation of ADAM17 in mice (Brandl et al., 2010; Chalaris et al., 2010; Hassemer et al., 2010). ADAM17 deficiency has been reported in three humans so far. These patients suffered from severe inflammatory skin and bowel disease (Blaydon et al., 2011; Tsukerman et al., 2015). One patient remained alive at the time of the report and has “led a relatively normal life” (Blaydon et al., 2011).

A review by Scheller et al. in 2011 reported 76 putative substrates of ADAM17 (Scheller et al., 2011), which continues to increase, though only a handful of these substrates have been further verified *in vivo*. ADAM17 typically cleaves its substrates in a *cis* manner and an examination of the cleavage site of various ADAM17 substrates reveals no strict consensus sequence, consistent with the sheddase's promiscuous activity. Proteomic studies of ADAM17 cleavage site specificities have, however, revealed a high preference for alanine, leucine, and valine residues, and a low preference for a proline residue (Caescu et al., 2009; Thorp et al., 2011; Tucher et al., 2014). Indeed, a proline residue engineered into the cleavage site of the ADAM17 substrates CD16a, CD16b, and L-selectin completely abrogate their shedding (Zhao et al., 2001; Jing et al., 2015). Despite ADAM17's relaxed sequence specificity, the sheddase tends to require a cleavage region with an α-helical conformation and appropriate physical length (Kishimoto et al., 1995; Mezyk et al., 2003; Stawikowska et al., 2013). The specific site of cleavage may also depend on the type of membrane linkage by the substrate. For instance, the human IgG Fc receptor CD16a (FcγRIIIa) is a transmembrane protein and CD16b (FcγRIIIb) is GPI-linked to the plasma membrane. These substrates have identical cleavage regions, yet CD16a is cleaved at a single location (Lajoie et al., 2014; Jing et al., 2015), whereas CD16b is cleaved at three locations in close proximity (Galon et al., 1998; Jing et al., 2015).

Ectodomain shedding by ADAM17 is regulated in various manners, including gene expression, spatial redistribution of the sheddase and its substrates within the plasma membrane, proenzyme conversion, enzyme inhibition, and by allosteric control. The influences of these regulatory events differ per cell type, stimulus, and substrate. An interesting feature of ADAM17 compared to other ADAM family members is that its sheddase activity is greatly increased upon cell activation (Edwards et al., 2008; Gooz, 2010; Matthews et al., 2016). An example of the rate and efficiency of this process is demonstrated by L-selectin shedding. Resting neutrophils express from 50,000 to 100,000 L-selectin molecules on their surface and essentially all are cleaved within minutes of neutrophil activation (Kishimoto et al., 1989; Walcheck et al., 1996a). Heterogeneous stimuli induce ectodomain shedding in diverse cell types, and this is primarily mediated by serine and threonine kinase-dependent intracellular signaling pathways (Gechtman et al., 1999; Díaz-Rodríguez et al., 2002; Soond et al., 2005; Schwarz et al., 2014), including PKC and MAPKs in neutrophils (Fan and Derynck, 1999; Rizoli et al., 1999; Alexander et al., 2000; Wang et al., 2011). ADAM17 sheddase activity is also increased during neutrophil apoptosis (Walcheck et al., 2006; Chalaris et al., 2007; Wang et al., 2010, 2013), and this process required caspases and mitochondrial reactive oxygen species (Wang et al., 2011). An area of active debate is the proximal target(s) of the intracellular signals and how they affect ectodomain shedding by ADAM17.

Though numerous mechanisms by which ADAM17 sheddase activity is increased upon cell activation have been described, a predominant theme is that intracellular signaling induces changes in the intrinsic activity of ADAM17. Conformational changes in ADAM17 upon cell activation are apparent by the exposure of binding sites for small molecule inhibitors and antibodies (Le Gall et al., 2010; Willems et al., 2010). This may involve phosphorylation of ADAM17's cytoplasmic region, which occurs following cell activation by various stimuli (Díaz-Rodríguez et al., 2002; Soond et al., 2005; Schwarz et al., 2014). Such a means of induction, however, is confounded by several studies showing that the cytoplasmic region of ADAM17 is not required for its sheddase activity (Reddy et al., 2000; Wang et al., 2009; Le Gall et al., 2010; Schwarz et al., 2013). However, the cytoplasmic region of ADAM17 may participate in a negative regulatory process. Xu et al. reported that ADAM17 in resting cells forms dimers in the cell membrane that associate with tissue inhibitor of metalloproteinase 3 (TIMP3) (Xu et al., 2012), which forms a non-covalent complex with the catalytic region of ADAM17 and blocks its activity (Amour et al., 1998; Smookler et al., 2006; Wisniewska et al., 2008). The cytoplasmic region of ADAM17 has been shown to be critical for dimer formation, and cell activation and MAPK activity were associated with ADAM17 dimer conversion to monomers and TIMP3 dissociation (Xu et al., 2012). Other protein partners with ADAM17 include two inactive members of the Rhomboid family, iRhom 1 and 2, which control ADAM17 maturation and trafficking to the cell surface (Adrain et al., 2012; Mcilwain et al., 2012; Li et al., 2015). Interesting is that iRhom2 expression is restricted to hematopoietic cells, whereas iRhom1 is more widely expressed (Christova et al., 2013), but not in leukocytes (Issuree et al., 2013). The iRhoms have been proposed to also play a role in the induction of ADAM17 sheddase activity upon cell activation (Maretzky et al., 2013; Lorenzen et al., 2016). Intracellular stores of ADAM17 occur in certain cells (Doedens and Black, 2000; Schlöndorff et al., 2000), and through a process facilitated by the iRhoms, Lorenzen et al. reported that ADAM17 surface expression can rapidly increase upon overt cell activation with a phorbol ester (Lorenzen et al., 2016). However, the importance of rapid ADAM17 upregulation as a general inducer mechanism of ectodomain shedding is an area of debate since this was not observed with physiological stimuli (Walcheck et al., 2006; Lorenzen et al., 2016), or in various cells activated with phorbol esters (Doedens and Black, 2000; Doedens et al., 2003; Horiuchi et al., 2007b).

The disintegrin-like and cysteine-rich domains of ADAM17 also modulate its sheddase activity (Reddy et al., 2000; Gonzales et al., 2004; Wang et al., 2009; Willems et al., 2010; Düsterhöft et al., 2013; Sommer et al., 2016). These domains contain cysteine residues that provide strictly conserved disulfide bonds (Takeda, 2016). ADAM17 has two highly conserved cysteine-X-X-cysteine sequences (CXXC, where XX represents two other amino acids), one located in the disintegrin-like domain and the other in the cysteine-rich domain (Figure 2) (Wang et al., 2009). Site-directed mutagenesis revealed that these regions are critical for ADAM17 activity (Wang et al., 2009). Similarly, within the β-subunit of integrin adhesion proteins are cysteine-rich regions that contain CXXC sequences, and this motif has been reported to be an active site for the modification of allosteric disulfide bonds and rapid conformational switches (O'neill et al., 2000; Walsh et al., 2004; Xu et al., 2016). Interesting is that sulfhydryl-modifying agents are known to alter L-selectin shedding by human neutrophils. For instance, the reducing agent DTT inhibited L-selectin shedding, whereas the oxidizing agent H_2_O_2_ induced its shedding (Lynam et al., 1996; Bennett et al., 2000; Wang et al., 2009). ADAM17 sheddase activity can also be directly modified by redox agents in a cell free assay (Wang et al., 2009). These findings suggest that ADAM17 is an allosterically regulatable enzyme, which perhaps occurs by thiol isomerases (Wang et al., 2009; Willems et al., 2010; Düsterhöft et al., 2013). Another motif in ADAM17 that may regulate its conformation and enzymatic activity is a cluster of cationic amino acids located in the membrane proximal region of the cysteine-rich domain of the sheddase (Figure 2). Upon cell activation and apoptosis, cell surface exposure of negatively charged, membrane phosphatidylserine may interact with the cationic amino acids and in turn increase the proximity of ADAM17's catalytic region with certain substrates (Sommer et al., 2016).

## Regulation of neutrophil recruitment by ADAM17

Neutrophils and endothelial cells constitutively express ADAM17 on their cell surface (Walcheck et al., 2006; Koenen et al., 2009; Weskamp et al., 2010). In contrast to global ADAM17 inactivation, conditional ADAM17 knockout mice that lack ADAM17 in myeloid cells, all leukocytes, or endothelial cells are viable and lack any obvious developmental abnormalities (Horiuchi et al., 2007a; Long et al., 2010, 2012; Weskamp et al., 2010; Arndt et al., 2011; Dreymueller et al., 2012; Mishra et al., 2016). Interesting is that either conditional ADAM17 knockout mice or hematopoietic chimeric mice that lacked ADAM17 in leukocytes demonstrated accelerated neutrophil recruitment at sites of sterile inflammation as well as infection (Long et al., 2010, 2012; Arndt et al., 2011; Tang et al., 2011; Mishra et al., 2015, 2016). This was also observed in mice receiving short-term treatment with an ADAM17 inhibitor (Tang et al., 2011; Mishra et al., 2015), demonstrating that the neutrophil recruitment pattern was not a developmental effect. One mechanism accounting for the accelerated recruitment of neutrophils is the disruption of L-selectin shedding (Tang et al., 2011; Long et al., 2012), which enhanced neutrophil tethering to L-selectin ligands on the vascular endothelium (Tang et al., 2011). CXCR2 surface levels on mouse and human neutrophils are also regulated by ADAM17 (Mishra et al., 2015). It is well-described that this chemokine receptor undergoes a rapid downregulation in expression by internalization upon binding its chemokine ligands, which is a reversible process since the receptor can be recycled back to the cell surface to bind additional ligands (Stillie et al., 2009). CXCR2 is also downregulated following overt neutrophil activation by non-ligand stimuli, including various PAMPs (Khandaker et al., 1998, 1999; Doroshenko et al., 2002; Mishra et al., 2015). This process involves ADAM17 and does not result in a recycling pool of CXCR2 (Mishra et al., 2015). Relevant to human neutrophils is that CD16b, an ADAM17 substrate described above (Wang et al., 2013; Jing et al., 2015), is also known to facilitate neutrophil attachment and migration through the vascular wall at sites of inflammation (Tsuboi et al., 2008). Various adhesion molecules expressed by endothelial cells and platelets that regulate hemostasis, barrier function, and leukocyte transmigration are also substrates of ADAM17, including GPIbα, GPV, JAMA, ICAM1, PECAM1, and VCAM1 (Garton et al., 2003; Bergmeier et al., 2004; Brill et al., 2009; Koenen et al., 2009; Weskamp et al., 2010). Taken together, ADAM17 can regulate different aspects of the multi-step process by which circulating neutrophils infiltrate inflamed tissue sites.

## ADAM17 activity during sepsis

Several lines of evidence from animal models and patients indicate aberrant ADAM17 activity during sepsis. Indeed, ADAM17 upregulation on the surface of circulating neutrophils was found to correlate with sepsis severity and patient outcome (Kermarrec et al., 2005). A recent study has also provided evidence that ADAM17 promoter polymorphism rs12692386 is a functional variant associated with the progression of sepsis severity (Shao et al., 2016). Patients with this polymorphism demonstrated an upregulation in ADAM17 expression and serum levels of several of its proinflammatory substrates (Shao et al., 2016). It has been reported that the plasma levels of several leukocyte- and endothelial cell-expressed, ADAM17 substrates are significantly elevated during sepsis, including L-selectin, ICAM-1, VCAM-1, CD16b, TNFα, IL-6R, TNFRI, and TNFRII, and some of these substrates demonstrated a positive correlation with disease severity (Ertel et al., 1994; Muller Kobold et al., 1998; Schulte et al., 2013; Zonneveld et al., 2014; Lerman and Kim, 2015). These adhesion proteins, receptors, and cell activating factors have a direct or indirect role in regulating neutrophil recruitment at sites of bacterial infection. Moreover, CXCR2 on the surface of circulating neutrophils is significantly downregulated during experimental sepsis and in human patients (Alves-Filho et al., 2009, 2010a).

Targeting leukocyte ADAM17 in animal models has been shown to greatly reduce damaging inflammation. For instance, ADAM17 inactivation in leukocytes significantly reduced tissue and plasma levels of proinflammatory factors and organ damage in localized and systemic endotoxemia models, in part, due to a marked reduction in TNFα levels and downstream effectors (Horiuchi et al., 2007a; Arndt et al., 2011). During *E. coli* infection, conditional ADAM17 knockout mice lacking ADAM17 in all leukocytes demonstrated a survival advantage and a marked reduction in bacterial levels at the site of infection (Long et al., 2010, 2012). In a model of polymicrobial sepsis, these conditional ADAM17 knockout mice also demonstrated enhanced survival, which corresponded with decreased bacteremia and levels of circulating proinflammatory cytokines, key determinants of sepsis severity (Mishra et al., 2016). Neutrophil recruitment at the site of infection was again found to be greatly increased in conditional ADAM17 knockout mice compared to control mice, and this likely accounted for the enhanced clearance of bacteria (Mishra et al., 2016).

## Concluding remarks

ADAM17 cleaves an assortment of type I and type II transmembrane proteins and GPI-anchored proteins at an extracellular site. Its sheddase activity is rapidly inducible and provides a mechanism for cells to respond very quickly to different environmental stimuli to reduce cell receptor densities. ADAM17 substrates on neutrophils and endothelial cells include L-selectin, CXCR2, CD16b, JAMA, ICAM1, PECAM1, and VCAM1, and the sheddase appears to function as a pivotal regulator of intravascular adhesion events (Figure 1A). It is well-established in animal models and by clinical evidence in humans that neutrophil recruitment at sites of infection is greatly impaired during the early stages of severe sepsis (Alves-Filho et al., 2010b; Sônego et al., 2014; Lerman and Kim, 2015). Sepsis may result in an over-induction of ADAM17 activity in neutrophils, endothelial cells, and other cells that in turn undermines the necessary balance between intravascular adhesion and de-adhesion events, and impairs neutrophil recruitment at the locus of infection (Figure 1B). Moreover, the ADAM17 substrate TNFα occurs at high levels in the blood during sepsis promoting neutrophil rigidity and the upregulation of integrin adhesion molecules, in turn causing occlusion of the microvasculature, ischemia, and tissue destruction through the release of cytotoxic factors (Brown et al., 2006; Alves-Filho et al., 2009; Lerman and Kim, 2015). Since there is not a strict consensus sequence at which ADAM17 cleaves, its fidelity may decrease during prolonged or excessive inflammation, resulting in more substrates and further cell dysfunction. In addition to aberrant ectodomain shedding during sepsis, various other mechanisms that underlie neutrophil dysfunction in the course of sepsis have been reported, as described in recent review articles (Sônego et al., 2014; Lerman and Kim, 2015; Zhang et al., 2016).

Despite years of active research, novel mechanistic insights about sepsis have not yet translated into effective host-directed drug treatments. Inflammation modulating research is shifting to therapeutic strategies to optimize the host's response to infection during sepsis. Therefore, it will be interesting to examine the targeting of ADAM17 as a host-directed therapeutic approach in patients. The potential benefits of ADAM17 inhibition on increasing neutrophil infiltration at sites of infection and reducing damaging inflammation may be exploited in clinical settings to reduce sepsis progression as well as its occurrence in high risk, general surgery patients. Of course, extrapolation of mouse model findings related to the effects of ADAM17 inactivation need to be confirmed in humans in which sepsis is a highly complex clinical syndrome. In addition, ADAM17-deficient mice are perinatal lethal (Peschon et al., 1998), mice expressing greatly reduced levels of ADAM17 demonstrate increased susceptibility to inflammatory diseases (Brandl et al., 2010; Chalaris et al., 2010), and loss-of-function mutations in ADAM17 cause inflammatory diseases in humans (Blaydon et al., 2011). In consideration of this, prolonged inhibition of ADAM17 could have detrimental consequences. However, pharmacological inhibitors of ADAM17 have advanced in specificity and progressed to clinical trials for cancer (for example, https://clinicaltrials.gov/ct2/show/record/NCT02141451), and have been reported to be well-tolerated (Friedman et al., 2009; Duffy et al., 2011). Thus, temporarily targeting ADAM17 for sepsis with highly specific inhibitors may not result in significant adverse effects. Moreover, it may be possible to selectively prevent the shedding of critical ADAM17 substrates that regulate leukocyte recruitment expressed by neutrophils, platelets, or endothelial cells by targeting their cleavage regions, which tend to vary between ADAM17 substrates, and in turn more precisely modulate leukocyte interactions with the vascular endothelium during sepsis.

## Author contributions

HM, JM, and BW: Substantial contributions to the conception, design, and drafting of the work; final approval of the version to be published; and agreement to be accountable for all aspects of the work.

## Funding

Research in the authors' laboratory is funded by the National Institutes of Health, including the current grants HL128580 and AI107543.

### Conflict of interest statement

The authors declare that the research was conducted in the absence of any commercial or financial relationships that could be construed as a potential conflict of interest.
